# Functional Comparison of Induced Pluripotent Stem Cell- and Blood-Derived GPIIbIIIa Deficient Platelets

**DOI:** 10.1371/journal.pone.0115978

**Published:** 2015-01-21

**Authors:** Mathias Orban, Alexander Goedel, Jessica Haas, Kirstin Sandrock-Lang, Florian Gärtner, Christian Billy Jung, Barbara Zieger, Elvira Parrotta, Karin Kurnik, Daniel Sinnecker, Gerhard Wanner, Karl-Ludwig Laugwitz, Steffen Massberg, Alessandra Moretti

**Affiliations:** 1 Medizinische Klinik und Poliklinik I, Klinikum der Universität München, Ludwig-Maximillians-Universität, Munich, Germany; 2 I. Medizinische Klinik und Poliklinik, Klinikum rechts der Isar der Technischen Universität München, Munich, Germany; 3 Department of Pediatrics and Adolescent Medicine, University Medical Center Freiburg, Freiburg, Germany; 4 Department of Experimental and Clinical Medicine, University of Magna Graecia, Medical School, Catanzaro, Italy; 5 Paediatric Haemophilia Centre, Dr. von Hauner Children’s Hospital, Ludwig-Maximillians-Universität, Munich, Germany; 6 Ultrastructural Research, Department Biology I, Biozentrum, Ludwig-Maximillians-Universität, Planegg-Martinsried, Germany; 7 DZHK (German Centre for Cardiovascular Research)—partner site Munich Heart Alliance, Munich, Germany; Johns Hopkins Univ. School of Medicine, UNITED STATES OF AMERICA

## Abstract

Human induced pluripotent stem cells (hiPSCs) represent a versatile tool to model genetic diseases and are a potential source for cell transfusion therapies. However, it remains elusive to which extent patient-specific hiPSC-derived cells functionally resemble their native counterparts. Here, we generated a hiPSC model of the primary platelet disease Glanzmann thrombasthenia (GT), characterized by dysfunction of the integrin receptor GPIIbIIIa, and compared side-by-side healthy and diseased hiPSC-derived platelets with peripheral blood platelets. Both GT-hiPSC-derived platelets and their peripheral blood equivalents showed absence of membrane expression of GPIIbIIIa, a reduction of PAC-1 binding, surface spreading and adherence to fibrinogen. We demonstrated that GT-hiPSC-derived platelets recapitulate molecular and functional aspects of the disease and show comparable behavior to their native counterparts encouraging the further use of hiPSC-based disease models as well as the transition towards a clinical application.

## Introduction

Glanzmann thrombasthenia (GT) is an autosomal recessive platelet disorder caused by mutations in the *ITGA2B* or *ITGB3* genes encoding the plasma membrane glycoproteins αIIb (GPIIb, CD41) and β3 (GPIIIa, CD61), respectively. GT is characterized by quantitative and qualitative defects in the αIIbβ3 integrin (CD41/CD61 complex), the main receptor for fibrinogen [1,2]. Fibrinogen binding to αIIbβ3 on activated platelets is critically involved in physiological hemostasis and pathological arterial thrombosis by promoting platelet interconnection and clot retraction [3,4]. Aggregation failure of GT platelets results in a lifelong bleeding tendency frequently requiring platelet transfusions [1].

Methods for *in vitro* generation of human platelets and megakaryocytes (MKs) have been established on the basis of bone marrow- or cord blood-derived CD34+ cells [5–7], which have been used to generate human models of GT [8]. Recently, other cell types have proven to be a source of thrombopoiesis *in vitro*. Conversion of fibroblasts into the megakaryocytic lineage by overexpression of transcription factors like OCT4 [9] or p45NF-E2 [10] in addition to cytokine treatment has been reported. Also mesenchymal stromal cells can be turned into MKs and platelets by overexpression of several Hox genes [11]. Over the past years, induced pluripotent stem cells (hiPSCs) are creating exciting opportunities for biomedical research [12,13]. Models of blood disorders based on hiPSCs have been generated for congenital anemias [14,15], myeloproliferative diseases [16], bone marrow failure [17] or complex hematopoietic dysfunctions associated with congenital syndromes [18]. These models were used as platforms to investigate mechanisms of genetic disease pathogenesis and provided new insights beyond existing disease modeling.

In addition to *in vitro* disease modeling, hiPSC-based strategies hold promises for novel therapeutic approaches in drug development and cell therapy [19]. In the last years, significant efforts have been made in finding more efficient ways for embryonic stem cell- and hiPSC-derived MK and platelet generation [20]. Recently, Sullivan et al. [21] reported on a hiPSC-based approach for generating megakaryocytes from patients suffering from GT and successful transgene expression restoring CD41/CD61 (αIIbβ3) complex surface expression on megakaryocytes.

However, before progress into clinical applications is possible, an emerging general issue of hiPSC-based models needs to be addressed: the comparability of hiPSC-derived cells and their native counterparts. So far, no systematic side-by-side comparison of hiPSC-derived blood lineages with terminally differentiated patient cell equivalents has been performed in terms of their characteristic pathophysiologic features. Earlier studies have shown that most of the hiPSC derivatives present properties that more closely resemble those of embryonic/postnatal tissues and hence do often not fully recapitulate the expected adult disease phenotype [22]. Recently, advances in culture conditions have enabled the generation of healthy hiPSC-derived oligodendrocytes and motor neurons, which reproduce features of adult human cells with a high degree of fidelity [23,24]. Whether this is also true for other hiPSC-derived cell types and in the setting of human disease has still to be proven. Therefore, the aim of our study was to generate a hiPSC-based model of GT and stringently analyze to which extent the disease phenotype of peripheral blood platelets is recapitulated in patient-specific hiPSC-derived equivalents.

## Materials and Methods

### Ethics Statement

All of the experimental procedures for this project were approved by the ethical review board of the Technische Universität München (project number 2109/08, approved 23^rd^ June 2008). We obtained written informed consent from the participants. All investigations with human tissue conform to the principles outlined in the declaration of Helsinki.

### Generation of hiPSCs

HiPSCs were generated from peripheral blood T-lymphocytes by Sendai virus-mediated overexpression of OCT3/4, SOX2, KLF4 and cMYC [25]. 5.3×10^5^ T-lymphocytes of each individual were infected, yielding 6 control (CTR) and 3 GT clones, which where manually picked on the same day. Of these, 2 clones from each individual were further used in the study (S1 Fig.). Culture of hiPSCs was performed following standard protocols. Pluripotency of hiPSCs was assessed using RT-PCR of endogenous pluripotency genes, immunostaining of whole EBs for cTNT, FOXA2 and NGF-R (P75), and in-depth gene expression analysis with PluriTest [26,27].

### Genetic analysis

Genomic DNA was isolated from blood mixed with ethylenediaminetetraacetic acid (EDTA) using Blood & Cell Culture DNA Kit (Qiagen). Exons were amplified and the resulting PCR product was separated in a 1% agarose gel. The purified DNA fragments were directly sequenced using PCR primers (S1 Table) [28].

### Thrombopoietic differentiation of hiPSCs

According to a protocol previously established by Takayama et al. [29], hiPSC colonies were removed from MEF feeders using a dissociation buffer (0.25% trypsin, 1 mg/ml collagenase IV, 20% KSR, 1 mmol/l CaCl_2_ in PBS), transferred onto irradiated C3H10T1/2 cells and differentiated with IMDM medium containing 10 mg/l insulin, 5.5 mg/l transferrin, 6.7 mg/ml selenium, 2 mmol/l L-glutamine, 15% fetal bovine serum (all Gibco), 0.45 μmol/l α-monothioglycerol (Sigma-Aldrich), 50 μg/ml ascorbic acid (Sigma-Aldrich) and 20 ng/ml recombinant human vascular endothelial growth factor (Invitrogen). On day 15, hiPSC-Sacs were disrupted with a cell scraper, crushed with a pipette and passed through a 40 μm cell strainer (BD Falcon). The yielded cells were transferred onto irradiated C3H10T1/2 cells and cultured in the same medium without vascular endothelial growth factor containing 100 ng/ml human TPO (R&D), 50 ng/ml human SCF (R&D), and 25 U/ml heparin (Sigma-Aldrich). Medium was changed every 3 days. According to Takayama et al. [29], floating cells from days 24 to 30 were collected for platelet and MK analysis.

### Reverse transcription PCR and quantitative real-time PCR

Reverse transcription PCR was used to assay Sendai viral transgenes in T-lymphocytes and hiPSCs. Quantitative real-time PCR was used for the assessment of expression of pluripotency genes and lineage markers representative of the three embryonic germ layers.

### Flow Cytometry

Cells from supernatant of day 24 to 30 were spun down and the pellets were resuspended in 200 μl 1% BSA/PBS and stained with 7-AAD and fluorescence-conjugated antibodies (S2 Table) for 30 min at room temperature. For PAC-1 binding, cells resuspended in Tyrode’s buffer were treated with 5 μmol/l ADP (Sigma-Aldrich) and 2.8 μmol/l Thromboxane A_2_ (TXA_2_, Enzo) or 1 U/ml thrombin for 20 min at 37°C and then stained with CD42b and PAC-1 antibodies for 30 min at room temperature. Flow cytometry data were acquired using a Gallios flow cytometer (Beckman Coulter). HiPSC-derived platelet-like particles were identified by their forward- and sideward-scatter pattern comparable to peripheral blood platelets and surface expression of CD42b. Each experiment including hiPSC-derived platelets and MKs was performed with both CTR and GT-hiPSC lines with at least three replicates performed in each group if not otherwise stated.

### Immunofluorescence

Cytospin slides with cells from supernatant of day 24 to 30 were prepared for immunofluorescence studies. After attachment on Cytospin slides, cells were fixed in 2% paraformaldehyde/PBS or optionally permeabilized in 0.1% Triton X-100 and stained with 4′,6-diamidino-2-phenylindole (DAPI), conjugated CD41/CD61 and CD42b antibodies (S2 Table). Particles with a size of 3–10μm, staining positive for CD42b and negative for DAPI were considered platelet-like-particles. For cytoskeleton analysis cells from supernatant of day 24 to 30 were spun down and resuspended in Tyrode’s buffer and put into fibrinogen-coated wells with or without treatment with 5 μmol/l ADP and 2.4 μmol/l TXA_2_. After 20 minutes at room temperature cells were fixed and permeabilized as described above and stained with conjugated CD42b antibody and rhodamine-conjugated phalloidin labeling filamentous actin (F-actin). For quantification of fibrinogen binding, wells were coated with 100 μg/ml fibrinogen. Cells were stained with rhodamine and plated on fibrinogen-coated wells. After treatment with ADP and TXA_2_, wells were washed twice with PBS and attached cells were imaged with a DMI 6000B microscope (Leica). Each experiment including hiPSC-derived platelets and MKs was performed with both CTR and GT-hiPSC lines with at least three replicates performed in each group.

### Differential interference contrast microscopy

Cells from supernatant of day 24 to 30 were spun down and resuspended in Tyrode’s buffer. Differential interference contrast microscopy (DICM) images of platelets spreading on fibrinogen-coated wells after treatment with 5 μmol/l ADP and 2.4 μmol/l TXA_2_ were recorded using an inverted IX 70 microscope (Olympus). For all supporting movies, platelets and megakaryocytes spreading on fibrinogen-coated wells were imaged with DICM with either 40× (S1 to S3 Movies and S5 and S6 Movies) or 20× (S4 and S7 Movies) objective in the presence of ADP/TXA2. First image was taken after 1 min, subsequent images every minute. Time lapse movies of stacked images. All scale bars represent 10 μm. Representative movies for each sample.

### Transmission electron microscopy

Transmission electron microscopy (TEM) of samples prepared by chemical fixation was performed with an EM 912 transmission electron microscope (Zeiss) [30].

### Statistics

Data was expressed as mean ± SEM, when applicable. Groups were compared by two-tailed, unpaired t tests for values with a Gaussian distribution or by two-tailed, unpaired Mann-Whitney tests for values without a Gaussian distribution, considering P < 0.05 as significant.

## Results

### Generation and characterization of GT-specific hiPSCs from a patient carrying a novel compound heterozygous mutation in ITGA2B

**Figure 1:**
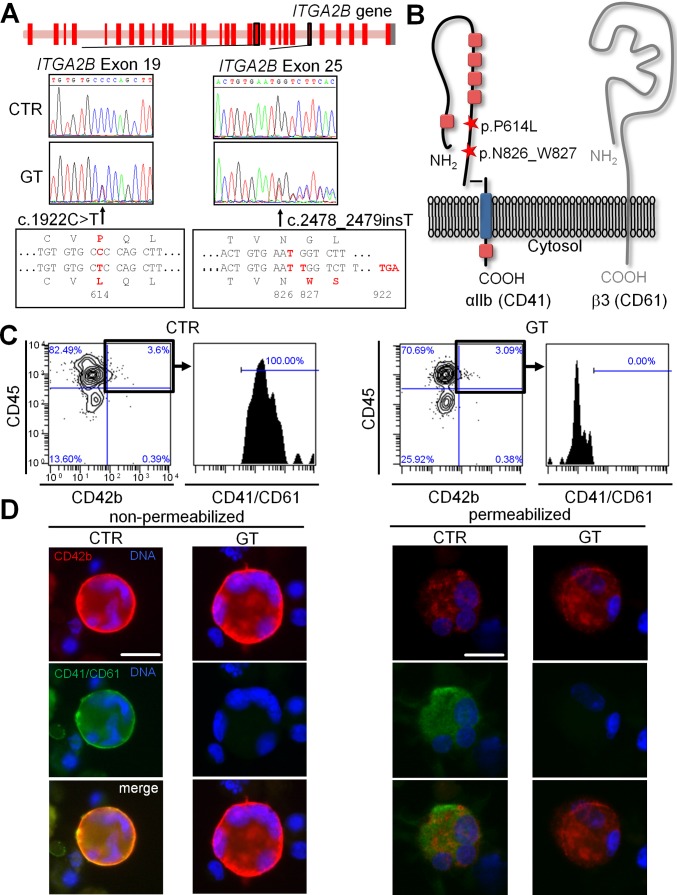
Characterization of hiPSC-derived MKs from a patient with a compound heterozygous mutation leading to Glanzmann thrombasthenia. (A) Scheme of the *ITGA2B* gene locus showing the location of the two mutations carried by the patient (GT) in exons 19 and 25. Sequencing data confirming the point mutation 1922C>T on exon 19 and the insertion 2478_2479insT on exon 25. (B) Scheme of the GPIIbIIIa receptor showing the location of the two mutations. (C) Flow cytometry of CD45, CD42b and CD41/CD61 expression on hiPSC-derived MKs. Cells were stained with anti-CD45 (y-axis) and anti-CD42b (x-axis) antibodies. CD42b and CD45 double positive cells were gated and stained for CD41/CD61. For gating hierarchy see S10 Fig. (D) Immunofluorescence of hiPSC-derived MKs. Cells were stained with anti-CD42b (red), anti-CD41/CD61 (green) antibodies and DAPI (blue) with (right) or without (left) Triton-X100 permeabilization. All scale bars represent 20 μm. Representative images for each sample.

Genetic screening of an 18-year-old woman diagnosed with GT revealed a compound heterozygous genetic background with two novel mutations in the *ITGA2B* gene: C to T nucleotide substitution at position 1922 (exon 19) resulting in a P614L amino acid exchange; and single T insertion at position 2478 (exon 25) leading to a frameshift N826_W827 and a truncation of the αIIb protein (CD41) after amino acid 921 due to a premature stop codon (Fig.1A). Both mutations are located in the heavy chain of the extracellular part of αIIb, shortly before the transmembrane domain (Fig.1B).

Blood T-lymphocytes were isolated from the GT patient and a healthy control (CTR), expanded and reprogrammed to pluripotency using non-integrating Sendai viruses (S1A Fig.). From each donor, two independent hiPSC lines were selected and further characterized. The difference in persistence of Sendai viruses in the CTR- and GT-hiPSCs clones at passage 6 suggests emergence of clones from independent reprogramming events (S1B Fig.). RT-PCR demonstrated loss of Sendai viruses after passage 10 (S1C Fig.). All hiPSC lines showed human embryonic stem cell (hESC)-like morphology and marker expression (S1D and S1E Fig.). Pluripotency was confirmed by upregulation of endodermal, mesodermal and ectodermal markers upon embryoid-body differentiation as shown by qPCR (S2A Fig.). In addition immunofluorescence analysis of 26-days-old whole EBs demonstrated expression of FOXA2 (endoderm), cTNT (mesoderm), and P75 (ectoderm) confirming differentiation into cell types of all three germ layers at the protein level (S2B Fig.). Moreover, genome-wide gene expression profile of undifferentiated GT- and CTR-hiPSCs showed high similarity to pluripotent stem cells, as confirmed by PluriTest (S2C Fig.).

### Analysis of integrin expression in GT-hiPSC-derived megakaryocytes and platelets reveals lack of CD41/CD61 complex

Hematopoietic differentiation of GT- and CTR-hiPSCs led to the formation of hiPSC-Sacs containing CD34+ cells presumably representing hematopoietic progenitor cells (HPCs) (S3A-C Fig.) [31]. Further analysis of HPCs demonstrated the expression of CD41 in a subset of CTR cells, which likely represent earlier stages of HPCs [32]. CD34+ HPCs from GT lacked CD41/61 complex expression (S3D Fig.). The number and viability of hiPSC-derived CD34+ HPCs was comparable between CTR and GT (S3E Fig.). HPCs extracted from hiPSC-Sacs were further differentiated into cells resembling MKs, monocytes and lymphocytes (S4A Fig.). CTR- and GT-hiPSC-derived MKs attached to fibrinogen, with the latter showing slightly reduced surface spreading, as imaged with DICM and shown by phalloidin staining of actin filaments (S4B and S4C Fig.). We could not detect any difference in numbers, viability or polyploidy between CTR- and GT-hiPSC-derived MKs (S4D and S4E Fig.). Membrane expression of CD42b, CD45 and CD31 was comparable (Fig.1C and 1D, S4F Fig.). However, in GT-MKs the CD41/CD61 complex was detectable neither at the cell surface nor in the intracellular compartment (Fig.1C and 1D) using two different anti-CD41/CD61 antibodies (S5 Fig.), suggesting rapid degradation of the mutated protein. The specificity of both antibodies was confirmed using flow cytometry on peripheral blood platelets of CTR and GT (S6 Fig.).

**Figure 2:**
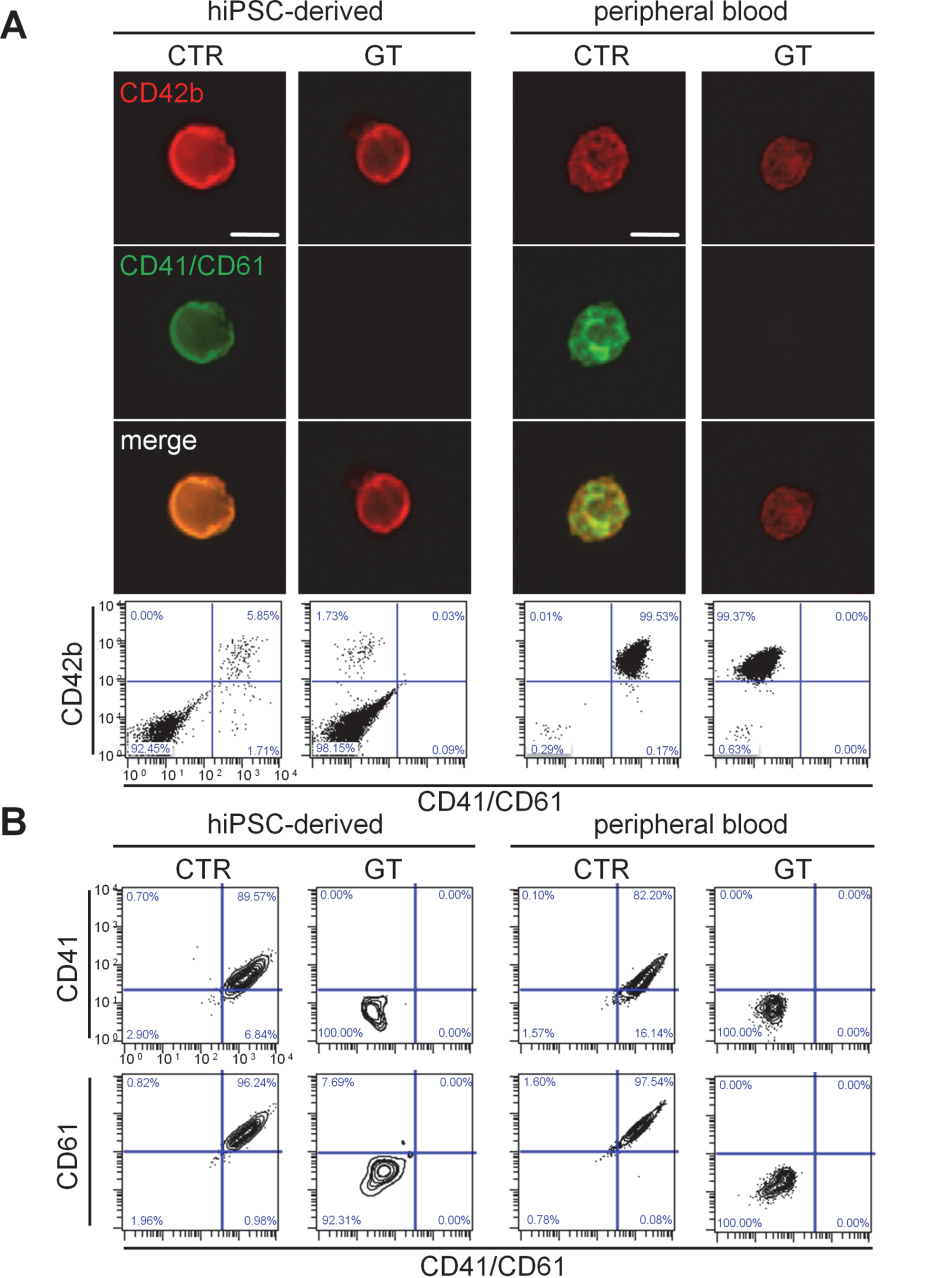
Generation and integrin surface expression of hiPSC-derived platelets from GT and CTR. (A) Immunofluorescence (top) and flow cytometry (bottom) of hiPSC-derived and peripheral blood platelets. The cells were stained with anti-CD42b (red / y-axis), anti-CD41/CD61 (green / x-axis) antibodies and DAPI (blue). (B) Flow cytometry of CD41 (GPIIb) and CD61 (GPIIIb) surface expression on hiPSC-derived and peripheral blood platelets. The cells were stained with anti-CD42b, anti-CD41/CD61, anti-CD41 and anti-CD61 antibodies. The FSC/SSC log gate of peripheral platelets was applied and further gated for CD42b+ cells. All scale bars represent 10 μm. Representative images for each sample.

Subsequently, we analyzed platelet-like particles generated from hiPSC-derived MKs. The criteria used for platelet identification are defined in the Material and Methods section. The average platelet yield of 3.8 platelets per hiPSC-derived MK was comparable to published data [29], with no significant difference between CTR and GT (S7A Fig.). TEM confirmed presence of granules and the open canalicular system in hiPSC-derived platelets of CTR and GT (S7B Fig.). When compared to CTR, GT-hiPSC-derived platelets exhibited equivalent expression of CD42b, while lacking CD41/CD61 complex (Fig. 2A, left panels). We excluded unspecific antibody binding to monocyte- or lymphocyte-derived platelet-sized particles by lack of CD14 or CD3 surface expression on hiPSC-derived platelets (S7C Fig.). Diseased and control platelets freshly isolated from blood displayed similar protein expression patterns (Fig. 2A, right panels). Moreover, using chain specific antibodies, further flow cytometry analysis revealed not only absence of CD41 (αIIb), as expected, but also of CD61 (β3) on GT-hiPSC-derived platelets (Fig. 2B, left panels). Platelets derived from peripheral blood showed the same distinctive features (Fig. 2B, right panels), suggesting that mutated CD41 protein affects surface expression of wild type CD61.

### GT-hiPSC-derived platelets exhibit similar functional defects as their blood counterparts: lack of agonist-induced activation, impaired surface spreading and adhesion, and abnormal cytoskeleton rearrangement

**Figure 3:**
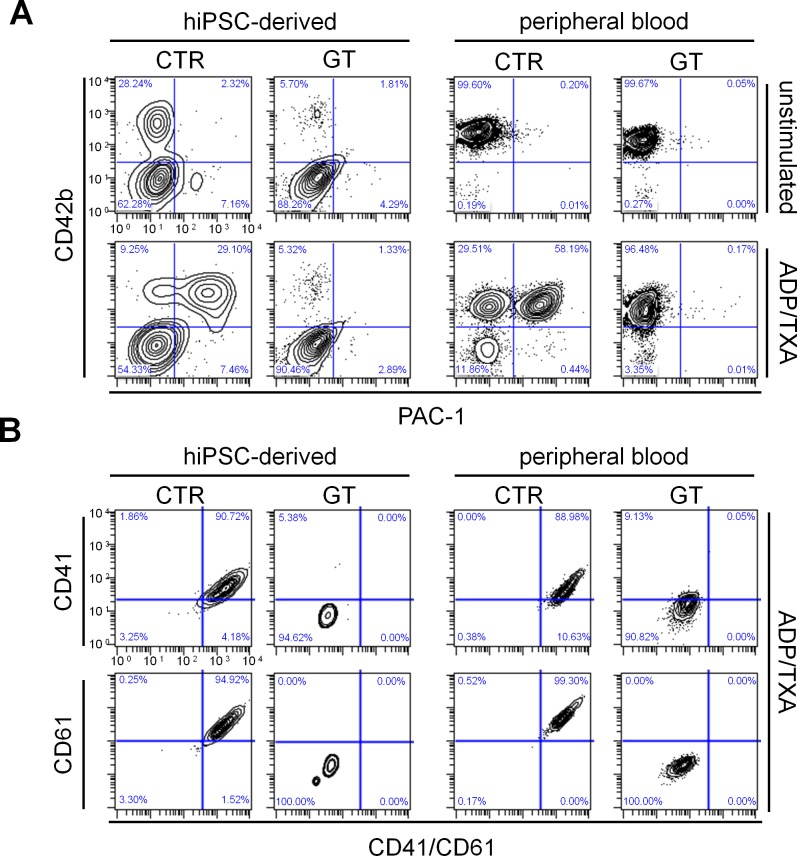
Flow cytometry of PAC-1 binding and integrin surface expression after platelet activation. (A) Cells were stained with PAC-1 (x-axis) and anti-CD42b (y-axis) antibodies in the absence (top) or presence (bottom) of ADP/TXA_2_. (B) Flow cytometry of CD41 (GPIIb) and CD61 (GPIIIa) surface expression on hiPSC-derived and peripheral blood platelets in the presence of ADP/TXA_2_. After activation with ADP/TXA_2_, cells were stained with anti-CD42b, anti-CD41/CD61, anti-CD41 and anti-CD61 antibodies. The FSC/SSC log gate of peripheral platelets was applied and further gated for CD42b+ cells.

To test integrin inside-out signaling *in vitro*, hiPSC-derived and peripheral blood platelets were stimulated with adenosine diphosphate and thromboxane A_2_ (ADP/TXA_2_) or thrombin and stained with PAC-1, which specifically detects only the activated conformation of αIIbβ3. GT platelets from both sources showed diminished PAC-1 binding after both agonist treatments compared to corresponding CTR cells (Fig. 3A and S8A Fig.). We did not observe any significant difference in PAC-1 binding between peripheral blood and hiPSC-derived platelets (S8B Fig.). Even after stimulation with ADP/TXA_2_, patient hiPSC-derived and peripheral blood platelets still lacked CD41, CD61 and CD41/CD61 complex expression, as revealed by flow cytometry analysis with chain specific antibodies (Fig. 3B). These results suggest that reduced PAC-1 binding is caused by an impaired complex formation, rather than reduced ligand affinity.

**Figure 4:**
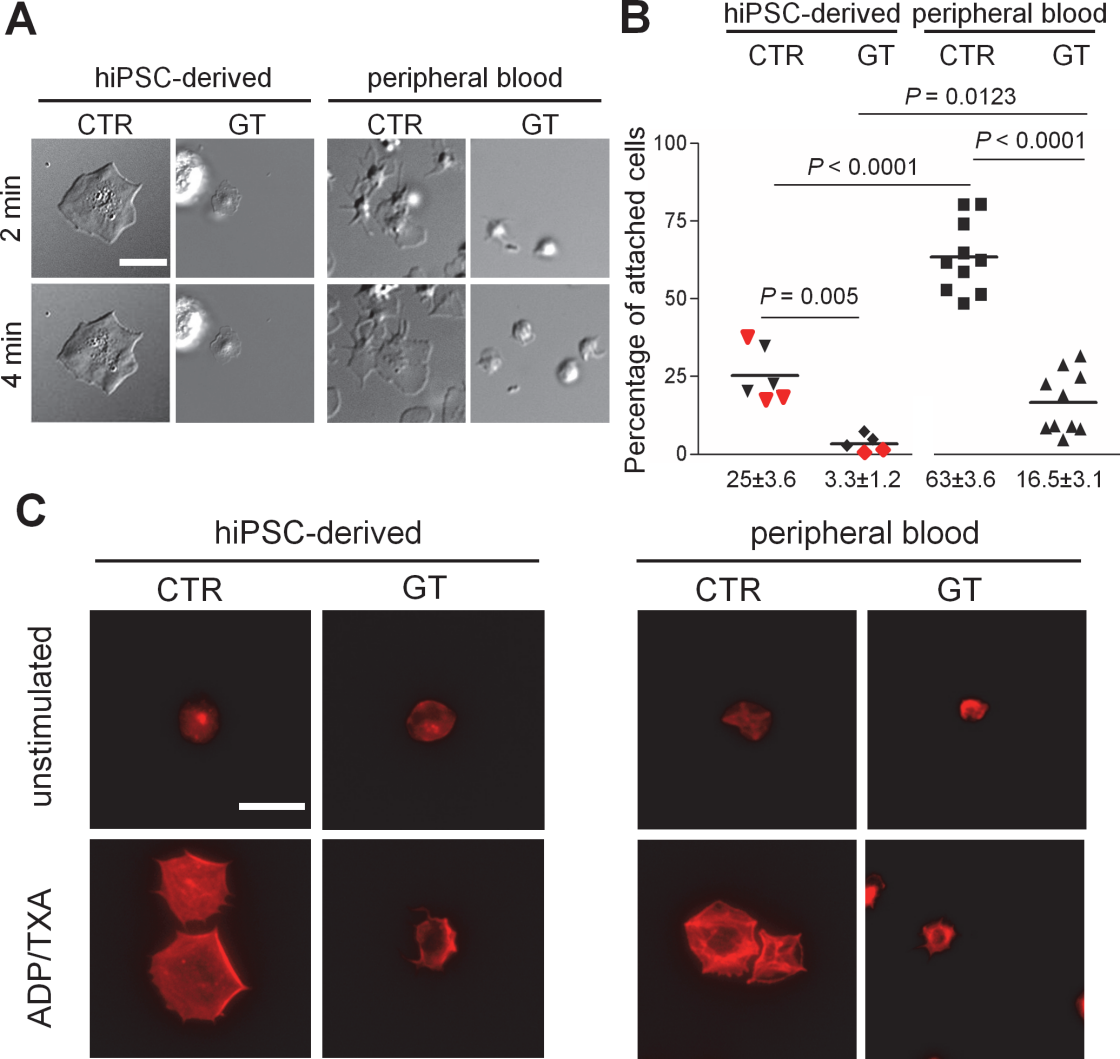
Surface spreading, adhesion and cytoskeleton rearrangement of peripheral blood and hiPSC-derived platelets. (A) Platelets spreading on fibrinogen-coated wells were imaged with DICM in the presence of ADP/TXA_2_ at the indicated time points. For complete movie sequence see S1, S4, S5, S7 Movies. (B) Quantification of fibrinogen binding of MKs and platelets. The percentages of attached cells are shown as scatter columns with horizontal bars representing the mean. In the hiPSC-derived groups, red and black symbols indicate results from the two hiPSC clones. A total mean of 1991±262.6 cells per sample was counted before washing. P-values from unpaired t-tests; peripheral blood platelets from GT (n = 10) and CTR (n = 10); hiPSC-derived platelets from GT (n = 5) and CTR (n = 6). (C) Rhodamine-conjugated phalloidin was used to stain actin filaments after permeabilization of platelets spreading on fibrinogen. All cells shown stained positive for CD42b and negative for DAPI. All scale bars represent 10 μm. Representative images for each sample.

Next, we examined platelet spreading on fibrinogen after ADP/TXA_2_ activation (Fig. 4). Healthy platelets from hiPSCs and peripheral blood formed filopodia and lamellipodia followed by rapid flattening on the fibrinogen matrix (Fig. 4A and S1–S4 Movies). In contrast, diseased platelets only attached loosely with subtle membrane movements; full-fledged lamellipodia formation or flattening was never observed (Fig. 4A and S5–S7 Movies). For quantification of platelet adherence to fibrinogen, we seeded rhodamine-stained cells from the culture supernatant onto fibrinogen-coated cover slides, activated the cells with ADP/TXA_2_ and counted adherent cells after a washing step, exposing them to shear. While approximately 25% of the CTR-hiPSC-derived cells remained attached, only 3% of GT-hiPSC-derived cells still adhered after washing. We obtained similar differences in platelet adherence between CTR and GT peripheral blood platelets, although the overall adherence rates of peripheral blood cells were markedly higher: 63% in the CTR and 16% in the GT groups, respectively (Fig. 4B). In order to investigate the possible reasons of such intra-individual difference between hiPSC-derived and peripheral blood platelets, we compared the amount of viable, and therefore functional, platelets between the two sources. Since shedding of CD42b has been reported to identify platelets with a necrotic phenotype *ex vivo* [33], we performed flow cytometry experiments using antibodies against CD42b and the CD41/CD61 complex (S9A Fig., left). In CTR peripheral blood platelets over 99% of all CD41/CD61+ cells co-stained positive for CD42b, indicating that platelet viability was preserved during the handling process. In contrast, we found a significantly reduced percentage of viable platelets (68%) in the CTR-hiPSC-derived group (S9B Fig.). As GT-hiPSC-derived platelets do not express CD41/CD61, we also assessed viability using CD31 (PECAM-1) as an alternative platelet marker (S9A Fig., right) [34]. We obtained similar percentages of viable cells in the CTR-hiPSC-derived samples (63%, S9B Fig., left column, and S9C, left column). Most importantly, we measured almost identical values in the GT-hiPSC-derived platelets (62%, S9C Fig.), suggesting that the hiPSC source is subject of more platelet degradation than peripheral blood, which may account, at least in part, for the observed differences in adhesion.

Finally, we investigated cytoskeleton remodeling processes by staining platelets spreaded on a fibrinogen-coated matrix with rhodamine-conjugated phalloidin, in order to visualize actin filament formation. After activation with ADP/TXA_2_, healthy CTR-hiPSC-derived and peripheral blood platelets displayed extensive cytoskeleton rearrangement, filopodia and lamellipodia formation as well as full flattening. In contrast, GT-hiPSC-derived and peripheral blood platelets showed filopodia, but lacked lamellipodia formation and full cytoskeleton organization (Fig. 4C). Indeed, it has been reported that GPIIbIIIa deficient platelets can undergo actin polymerization leading to filopodia and membrane ruffling, but fail to form lamellipodia [35], highlighting the important role of GPIIbIIIa for the morphologic changes during platelet activation and adherence to fibrinogen.

## Discussion

In this study we generated a patient-specific hiPSC-based model of Glanzmann thrombasthenia with compound heterozygosity of 2 novel mutations in the *ITGA2B* gene, which enabled the perpetual *in vitro* differentiation of GPIIbIIIa deficient platelets and MKs from hematopoietic precursors. Side-by-side comparison of healthy CTR- and GT-hiPSC-derived platelets with their peripheral blood counterparts demonstrated comparable molecular and functional features between cells of both sources, with similarly compromised integrin expression, PAC-1 binding, surface spreading and adhesion to fibrinogen in GT diseased cells.

Interestingly, both GT-hiPSC-derived and native platelets not only showed absence of the mutated CD41 (αIIb, GPIIb) protein but also presented a concurrent lack of CD61 (β3, GPIIIa) surface expression. This finding suggests that mutated αIIb subunit negatively affects wild type β3, likely by impaired complex formation in the rough endoplasmatic reticulum and subsequent degradation of both proteins, as already reported [1]. Moreover, it highlights the ability of hiPSC-based disease models to reproduce the molecular pathognomonic properties of native affected cells.

In our assay of platelet adhesion on fibrinogen, we measured an overall higher level of adhesion of freshly isolated blood platelets compared to hiPSC-derived cells. We can in part attribute this difference to a reduced viability of hiPSC-derived platelets in culture, as measured by shedding of CD42b in CD41/CD61+ or CD31+ positive platelets. In addition, the differences in platelet purity between the two sources might influence the adhesion rate: hiPSC-derived platelets isolated from cell culture supernatant cannot be completely separated from cell culture debris and have a higher degree of heterogeneity compared to thrombocytes in platelet rich plasma, leading to an underestimation of platelet adherence. Furthermore, it has been reported that hiPSC-derived cells are less mature than their native counterparts, which potentially could be another reason for lower platelet adhesion in the hiPSC-derived platelet group [22]. Nonetheless, both blood- and hiPSC-derived platelets confirmed the defective adhesion properties expected in GT.

The wide application of hiPSCs in disease modeling, drug screening and therapy is limited by the difficulty in comparing diseased hiPSC-derived cells with their native counterparts [36]. Taking advantage of the accessibility of blood cells, our study demonstrates for the first time that hiPSC-derived platelets of a patient with Glanzmann thrombasthenia recapitulate, to a certain degree, the disease phenotype of peripheral blood platelets on a molecular and functional level *in vitro*. Our data shows that hiPSC-derived cells bear sufficient, but not complete resemblance to their native counterparts, a finding that highlights the importance of our strategy of side-by-side comparison. This approach could serve as an example for evaluating hiPSCs and their differentiated derivatives to reveal both validity and limitation of their disease modeling capabilities. Likewise, our results encourage the use of hiPSC-based disease models, especially in settings where the native target cell is not readily available [37,38].

Despite a general bleeding disorder in GT patients, bleeding severity differs considerably between affected individuals, even within the same family or ethnic group [39]. Understanding the molecular basis leading to this high phenotypic variability is subject of intensive research [40]. However, the scattered geographic distribution of patients with rare platelet diseases such as Glanzmann thrombasthenia impedes frequent clinical contact and scientific workflow even in specialized centers. Generating a stable hiPSC-line from a single blood donation enables researchers to work constantly on pathophysiologic questions independent of these temporal or spatial constraints and can provide new insights on differences in disease severity among affected individuals.

Furthermore, hiPSCs allow for the continuous production of diseased MKs and their derivatives in culture, creating new possibilities for studying the ontogeny of megakaryopoiesis in health and disease [17]. In addition, harnessing hiPSC-derived models of platelet receptor defects may expand our knowledge of the pharmacological mechanisms of drugs targeting platelets, such as GPIIbIIIa inhibitors, as treatment for cardiovascular diseases [41]. Others studies have already demonstrated the utility of hiPSC-derived models for evaluating established pharmacotherapies or new drug combinations in rare diseases [42].

However, for a broader application of platelets and other cells derived from hiPSCs, the low efficiency and high variability of lineage-specific differentiation poses a substantial challenge [43]. Increasing maturity and number of desired cells could therefore pave the road for clinical applications of hiPSC-derived cells in general [44]. The platelet yield we obtained from CTR- and GT-hiPSCs in this study is insufficient for clinical applications and reflects the difficulties in generating patient-specific platelets *in vitro*. Few reports have described higher platelets yields using non-patient-specific hiPSC lines; however, the numbers still remain too low for transfusion therapies. Advancements in dynamic three-dimensional culture conditions mimicking bone marrow and donor-specific immortalized megakaryocytic lines, which are expandable over longer periods, have improved the efficiency of *in vitro* platelet generation [20,45]. This development could augment *in vitro* platelet numbers to clinically significant amounts. However, it still needs to be proven that these methods are applicable to patient-specific lines and thus can be used for personalized transfusions therapies in patients with known genetic platelets disorders or life-threating bleedings. This is of special interest for platelets, since anucleated cells circumvent the concern of tumorigenesis in recipients of stem cell therapies.

## Supporting Information

S1 Fig Reprogramming of GT patient T-lymphocytes to hiPSCs. (A) Representative images of PBMCs on day 0 (i) and on day 5 (ii) of activation in the presence of anti-CD3 antibody and IL-2. Generated hiPSC lines show normal hESC morphology (iii) and typical alkaline phosphatase activity (iv). Scale bars represent 100 μm. (B) Immunostaining of hiPSC clones at passage 6 for Sendai viral antigen (red) and co-staining with DAPI (blue). Scale bar represents 50 μm. (C) Reverse transcription PCR analysis confirms presence (lane 3) of Sendai viral transgenes in infected T lymphocytes (ITL) and loss (lane 4 and 5) in CTR- (left panel) and GT-hiPSC lines (right panel). TL indicates primary T lymphocytes. (D) Immunostaining of hiPSC clone for the pluripotency markers NANOG (red) and TRA1-81 (green) and co-staining with DAPI (blue). Scale bar represents 25 μm. (E) Quantitative real-time PCR analysis confirms upregulation of endogenous pluripotency genes (expression levels relative to T-lymphocytes). All expression values are normalized to GAPDH. (A) and (D) Representative images of GT-hiPSC colonies (clone 2). (B) Representative images for each sample.
(TIF)

S2 Fig Assessing pluripotency of hiPSCs. (A) Quantitative real-time PCR analysis confirms the potential of generated hiPSCs to differentiate into cells of all three germ layers (expression levels in embryoid bodies relative to respective hiPSC clone). All expression values are normalized to GAPDH. (B) Immunostaining of whole EBs (day 26) for FOXA2 (red, left), cTNT (red, middle) and P75 (NGF-R; green, right) and co-staining with DAPI (blue). Scale bar represents 25 μm. Representative images of CTR-hiPSCs (clone 2) (C) Gene expression analysis with PluriTest of undifferentiated GT- (clone 1) and CTR-hiPSCs (clone 1).
(TIF)

S3 Fig Generation of hiPSC-derived hematopoietic progenitor cells from GT and CTR. (A) Strategy used for differentiation of hiPSCs. (B) HiPSC-Sac formation at day 14–15 of hematopoietic differentiation (i) and higher magnification of the content of the hiPSC-Sac (ii) showing round cells resembling hematopoietic progenitors. Scale bars represent 200 μm. (C) and (D) Flow cytometry of CD34 (C) and CD41/CD61 (D) expression of hiPSC-derived cells within hiPSC-Sacs. HiPSC-derived cells within hiPSC-Sacs of GT and CTR were stained with anti-CD34 and anti-CD41/CD61 antibodies. The FSC/SSC log gate of large cells was applied. (E) Quantification of the percentage of CD34+ cells of total events (left, GT n = 5, CTR n = 8) and their viability (right, GT n = 3, CTR n = 4) measured as 7-AAD-negative cells by flow cytometry. P-values from unpaired t-tests. Representative images for each sample.
(TIF)

S4 Fig Generation of hiPSC-derived MKs from GT and CTR. (A) Hemacolor stain of GT- (right) and CTR- (left) hiPSC-derived cells. Arrows indicate cells with typical MK-like appearance. Lymphocyte-like cells and monocyte-like cells are marked by asterisks. (B) DIC microscopy images of GT- (right) and CTR- (left) hiPSC-derived MKs after activation with ADP/TXA_2_. Arrows indicate lamellipodia. Dashed lines mark cell body. (C) Rhodamine-conjugated phalloidin stain of GT- (right) and CTR- (left) hiPSC-derived MKs to selectively label actin filaments. Cells were stained after activation with ADP/TXA_2_. (D) Quantification of the percentage of MKs (CD42b and CD45 double positive cells) of all non-platelets (left, p-value from unpaired t-test) and their viability (right, p-value from unpaired Mann-Whitney test) measured as 7-AAD-negative cells by flow cytometry (GT n = 13, CTR n = 21). (E) Distribution of nuclei number per MK from DAPI staining assessed by fluorescence microscopy (total cells counted GT n = 159, CTR n = 169). (F) Flow cytometry of CD42b and CD31 expression on hiPSC-derived MKs. Cells were stained with anti-CD45, anti-CD42b (x-axis) and anti-CD31 (y-axis) antibodies. For gating hierarchy see S10 Fig. All scale bars represent 20 μm. Representative images for each sample.
(TIF)

S5 Fig Immunostaining of CD41/CD61 with two different antibodies of hiPSC-derived MKs from GT and CTR. Cells were stained with anti-CD42b (red) and two different anti-CD41/CD61 (green; antibody 1 = HIP8, antibody 2 = P2) antibodies and DAPI (blue) with (A) or without (B) Triton-X100 permeabilization. All scale bars represent 20 μm. Representative images for each sample.
(TIF)

S6 Fig Specificity of two different anti-CD41/CD61 antibodies assessed by flow cytometry. Peripheral blood platelets of CTR and GT were stained with anti-CD42b (y-axis) and two different anti-CD41/CD61 antibodies (x-axis; antibody 1 = clone HIP8; antibody 2 = clone P2) and DAPI (blue).
(TIF)

S7 Fig Generation of hiPSC-derived platelets from GT and CTR. (A) Quantification of hiPSC-derived platelet yield per MK from GT and CTR (GT n = 11, CTR n = 15) by flow cytometry. Total number of platelets was divided by the number of MKs. P-value from unpaired t-test (B) Transmission electron microscopy images of hiPSC-derived platelets from GT and CTR show granules (asterisks) and the open canalicular system (arrows). Scale bar represents 2 μm. (C) Flow cytometry of CD3 and CD14 expression of hiPSC-derived platelets. HiPSC-derived cells of GT and CTR were stained with anti-CD42b, anti-CD41/CD61, anti-CD45, anti-CD3 and anti-CD14 antibodies. The FSC/SSC log gate of peripheral platelets was applied. For gating hierarchy see S10 Fig. Representative images for each sample.
(TIF)

S8 Fig Flow cytometry of PAC-1 binding after platelet activation. (A) HiPSC-derived and peripheral blood platelets were stained with PAC-1 and anti-CD42b antibodies in the absence (top) and presence (bottom) of thrombin. The FSC/SSC log gate of peripheral platelets was applied and further gated for CD42b positive cells. Representative images for each sample. For gating hierarchy see S10 Fig. (B) Quantification of PAC-1 binding after activation with ADP/TXA_2_. HiPSC-derived and peripheral platelets of CTR and GT were stained with anti-CD42b and PAC-1 antibodies and exposed to ADP/TXA_2_. The percentage of PAC-1+ of all CD42b+ platelets after ADP/TXA_2_ exposure is shown. P-values from unpaired t-tests.
(TIF)

S9 Fig Assessment of platelet viability by flow cytometry. (A) HiPSC-derived platelets were stained with anti-CD42b (y-axis) and anti-CD41/CD61 or anti-CD31 (x-axis) antibodies. Continuous frames comprise either CD41/CD61+ or CD31+ platelets. Dashed frames comprise either CD42b and CD41/CD61 double positive or CD42b and CD31 double positive platelets. Representative images for each sample. For gating hierarchy see S10 Fig. (B) Quantification of the percentage of CD42b+ platelets of all CD41/CD61+ hiPSC-derived or peripheral blood platelets of CTR. (C) Quantification of the percentage of CD42b+ platelets of all CD31+ hiPSC-derived platelets of CTR and GT. P-values from unpaired t-tests.
(TIF)

S10 Fig Flow cytometry gating hierarchy of hiPSC-derived platelets and MKs. Flow cytometry gating hierarchy of hiPSC-derived MKs and platelets. Doublets of events were excluded with the FSC TOF (far left) and SSC TOF (left) parameters of the Gallios cytometer (Beckman Coulter). 7-AAD negative events (right) were further gated according to forward and sideward scatter parameters (far right). The FSC/SSC log gate of peripheral blood platelets was applied for analyzing platelets. A FSC high/SSC high population was selected for non-platelets or large cells including hiPSC-derived MKs and HPCs.
(TIF)

S1 Methods Supporting Material and Methods and Supporting References.
(DOCX)

S1 Movie Spreading of a CTR-hiPSC-derived platelet with lamellipodia formation.
(AVI)

S2 Movie Spreading of a CTR-hiPSC-derived platelet with filopodia formation.
(AVI)

S3 Movie Spreading of a CTR-hiPSC-derived platelet and megakaryocyte.
(AVI)

S4 Movie Spreading of CTR peripheral blood platelets.
(AVI)

S5 Movie Spreading of a GT-hiPSC-derived platelet.
(AVI)

S6 Movie Spreading of GT-hiPSC-derived platelets and megakaryocyte.
(AVI)

S7 Movie Spreading of GT peripheral blood platelets.
(AVI)

S1 Table PCR-Primers used for sequencing of exons containing the mutations in the *ITGA2B* gene.
(DOCX)

S2 Table Antibodies/Flow Cytometry Dye.
(DOCX)
