# Supplementary figures and images for: Functional Comparison of Induced Pluripotent Stem Cell- and Blood-Derived GPIIbIIIa Deficient Platelets
Source: PLoS One. 2015 Jan 21;10(1):e0115978. doi: 10.1371/journal.pone.0115978 (PMC4301811; doi:10.1371/journal.pone.0115978)

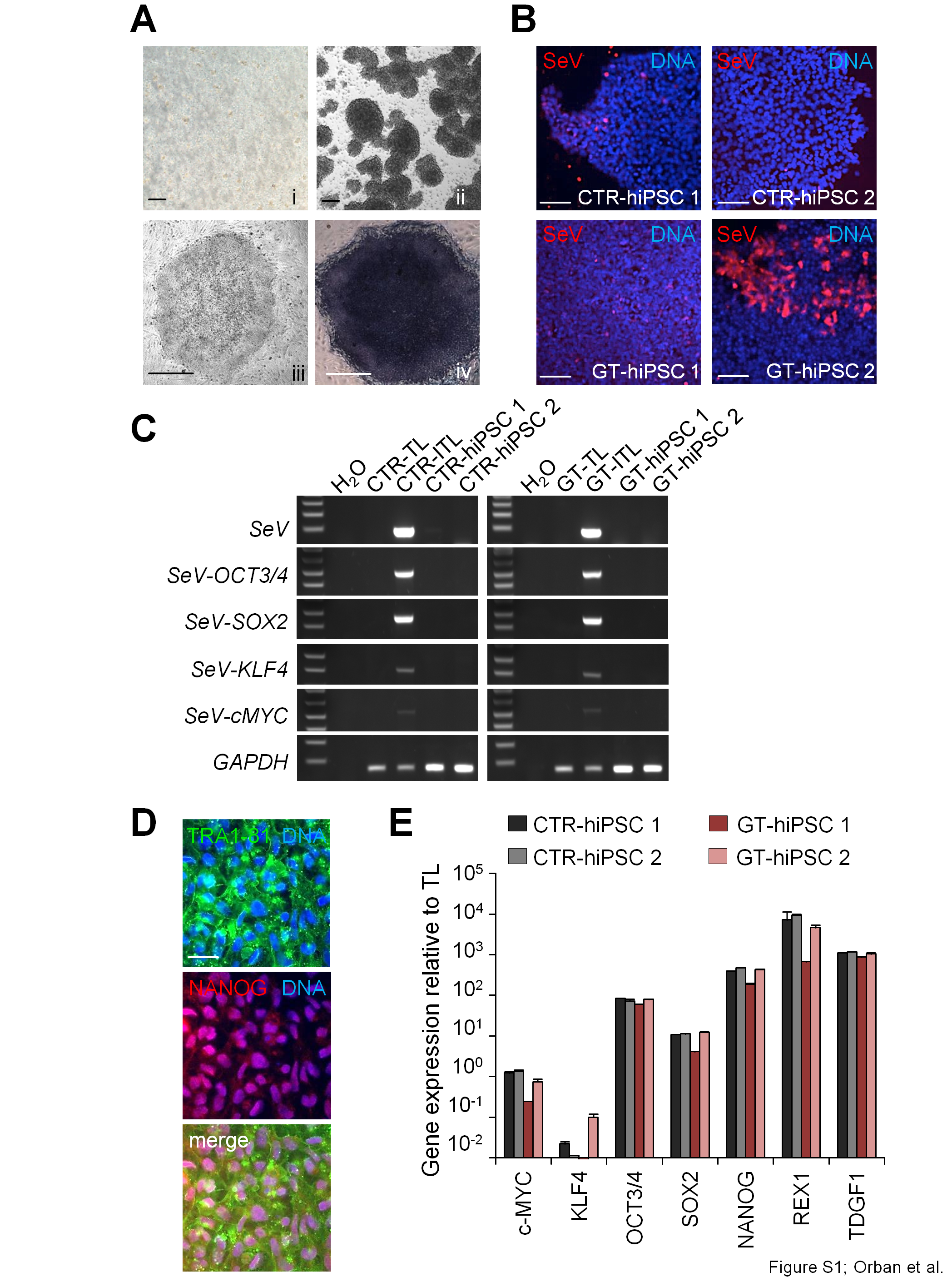

Supplement: S1 Fig — (A) Representative images of PBMCs on day 0 (i) and on day 5 (ii) of activation in the presence of anti-CD3 antibody and IL-2. Generated hiPSC lines show normal hESC morphology (iii) and typical alkaline phosphatase activity (iv). Scale bars represent 100 μm. (B) Immunostaining of hiPSC clones at passage 6 for Sendai viral antigen (red) and co-staining with DAPI (blue). Scale bar represents 50 μm. (C) Reverse transcription PCR analysis confirms presence (lane 3) of Sendai viral transgenes in infected T lymphocytes (ITL) and loss (lane 4 and 5) in CTR- (left panel) and GT-hiPSC lines (right panel). TL indicates primary T lymphocytes. (D) Immunostaining of hiPSC clone for the pluripotency markers NANOG (red) and TRA1-81 (green) and co-staining with DAPI (blue). Scale bar represents 25 μm. (E) Quantitative real-time PCR analysis confirms upregulation of endogenous pluripotency genes (expression levels relative to T-lymphocytes). All expression values are normalized to GAPDH. (A) and (D) Representative images of GT-hiPSC colonies (clone 2). (B) Representative images for each sample. (TIF) [file pone.0115978.s001.tif]

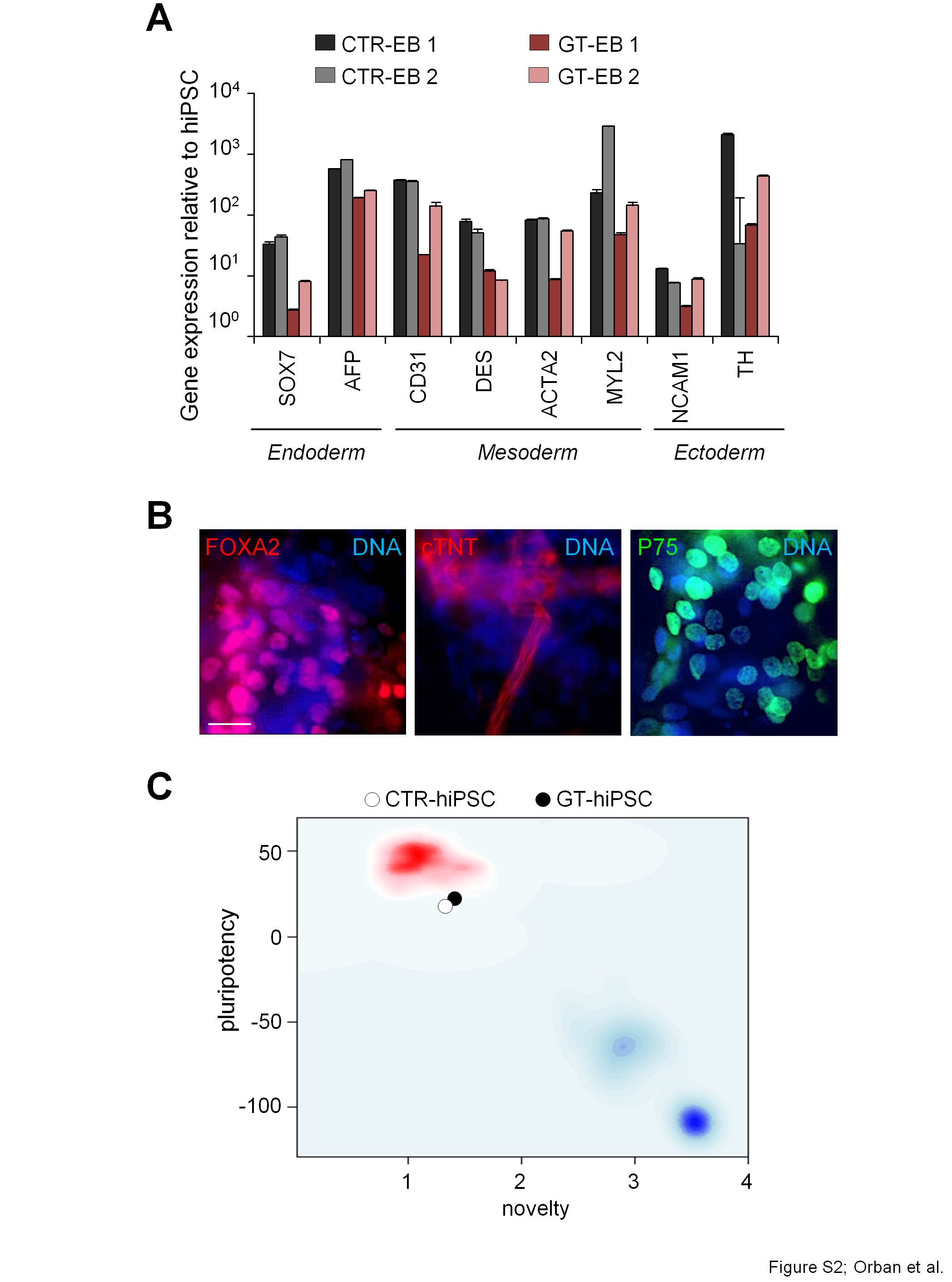

Supplement: S2 Fig — (A) Quantitative real-time PCR analysis confirms the potential of generated hiPSCs to differentiate into cells of all three germ layers (expression levels in embryoid bodies relative to respective hiPSC clone). All expression values are normalized to GAPDH. (B) Immunostaining of whole EBs (day 26) for FOXA2 (red, left), cTNT (red, middle) and P75 (NGF-R; green, right) and co-staining with DAPI (blue). Scale bar represents 25 μm. Representative images of CTR-hiPSCs (clone 2) (C) Gene expression analysis with PluriTest of undifferentiated GT- (clone 1) and CTR-hiPSCs (clone 1). (TIF) [file pone.0115978.s002.tif]

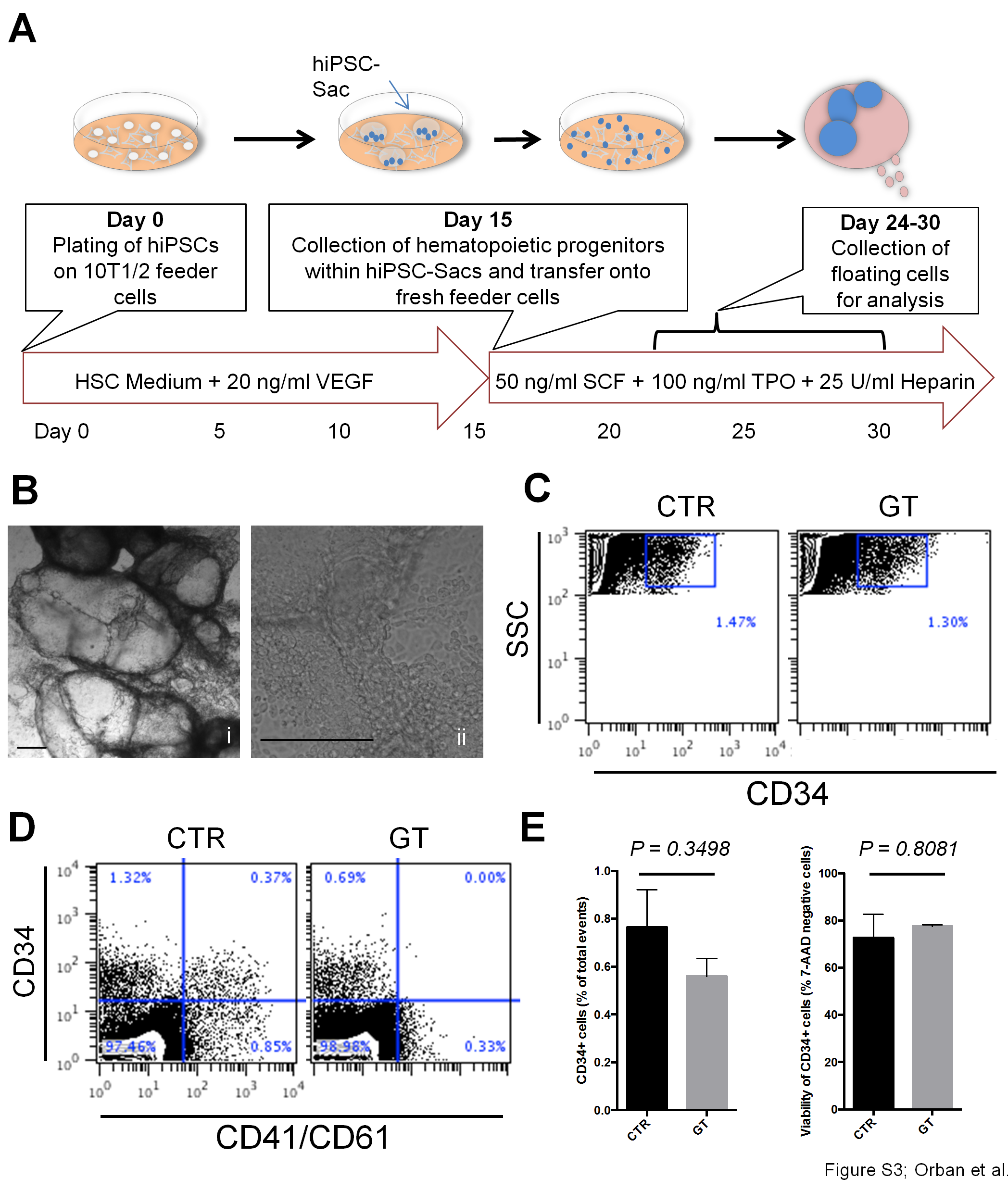

Supplement: S3 Fig — (A) Strategy used for differentiation of hiPSCs. (B) HiPSC-Sac formation at day 14–15 of hematopoietic differentiation (i) and higher magnification of the content of the hiPSC-Sac (ii) showing round cells resembling hematopoietic progenitors. Scale bars represent 200 μm. (C) and (D) Flow cytometry of CD34 (C) and CD41/CD61 (D) expression of hiPSC-derived cells within hiPSC-Sacs. HiPSC-derived cells within hiPSC-Sacs of GT and CTR were stained with anti-CD34 and anti-CD41/CD61 antibodies. The FSC/SSC log gate of large cells was applied. (E) Quantification of the percentage of CD34+ cells of total events (left, GT n = 5, CTR n = 8) and their viability (right, GT n = 3, CTR n = 4) measured as 7-AAD-negative cells by flow cytometry. P-values from unpaired t-tests. Representative images for each sample. (TIF) [file pone.0115978.s003.tif]

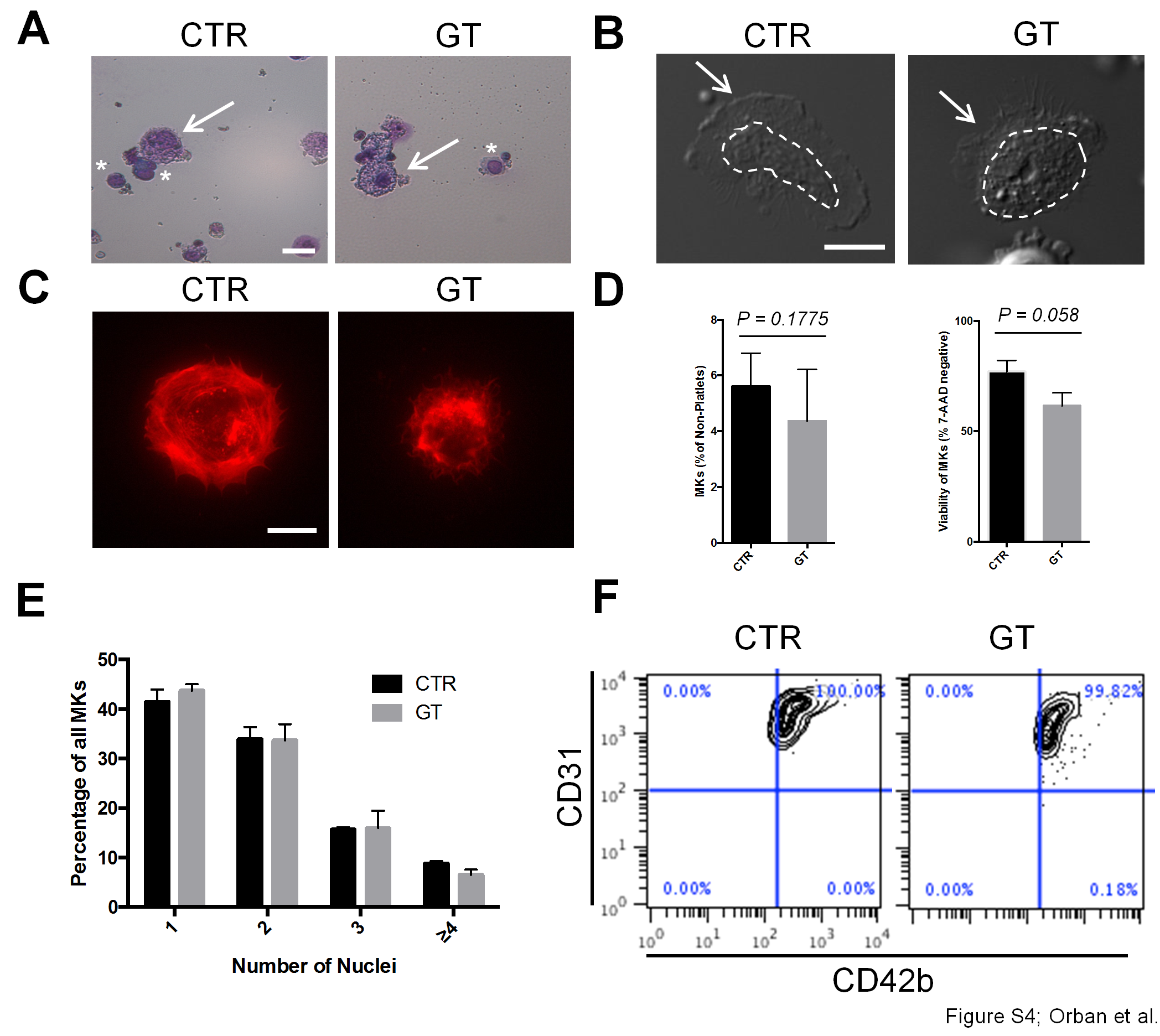

Supplement: S4 Fig — (A) Hemacolor stain of GT- (right) and CTR- (left) hiPSC-derived cells. Arrows indicate cells with typical MK-like appearance. Lymphocyte-like cells and monocyte-like cells are marked by asterisks. (B) DIC microscopy images of GT- (right) and CTR- (left) hiPSC-derived MKs after activation with ADP/TXA2. Arrows indicate lamellipodia. Dashed lines mark cell body. (C) Rhodamine-conjugated phalloidin stain of GT- (right) and CTR- (left) hiPSC-derived MKs to selectively label actin filaments. Cells were stained after activation with ADP/TXA2. (D) Quantification of the percentage of MKs (CD42b and CD45 double positive cells) of all non-platelets (left, p-value from unpaired t-test) and their viability (right, p-value from unpaired Mann-Whitney test) measured as 7-AAD-negative cells by flow cytometry (GT n = 13, CTR n = 21). (E) Distribution of nuclei number per MK from DAPI staining assessed by fluorescence microscopy (total cells counted GT n = 159, CTR n = 169). (F) Flow cytometry of CD42b and CD31 expression on hiPSC-derived MKs. Cells were stained with anti-CD45, anti-CD42b (x-axis) and anti-CD31 (y-axis) antibodies. For gating hierarchy see S10 Fig. All scale bars represent 20 μm. Representative images for each sample. (TIF) [file pone.0115978.s004.tif]

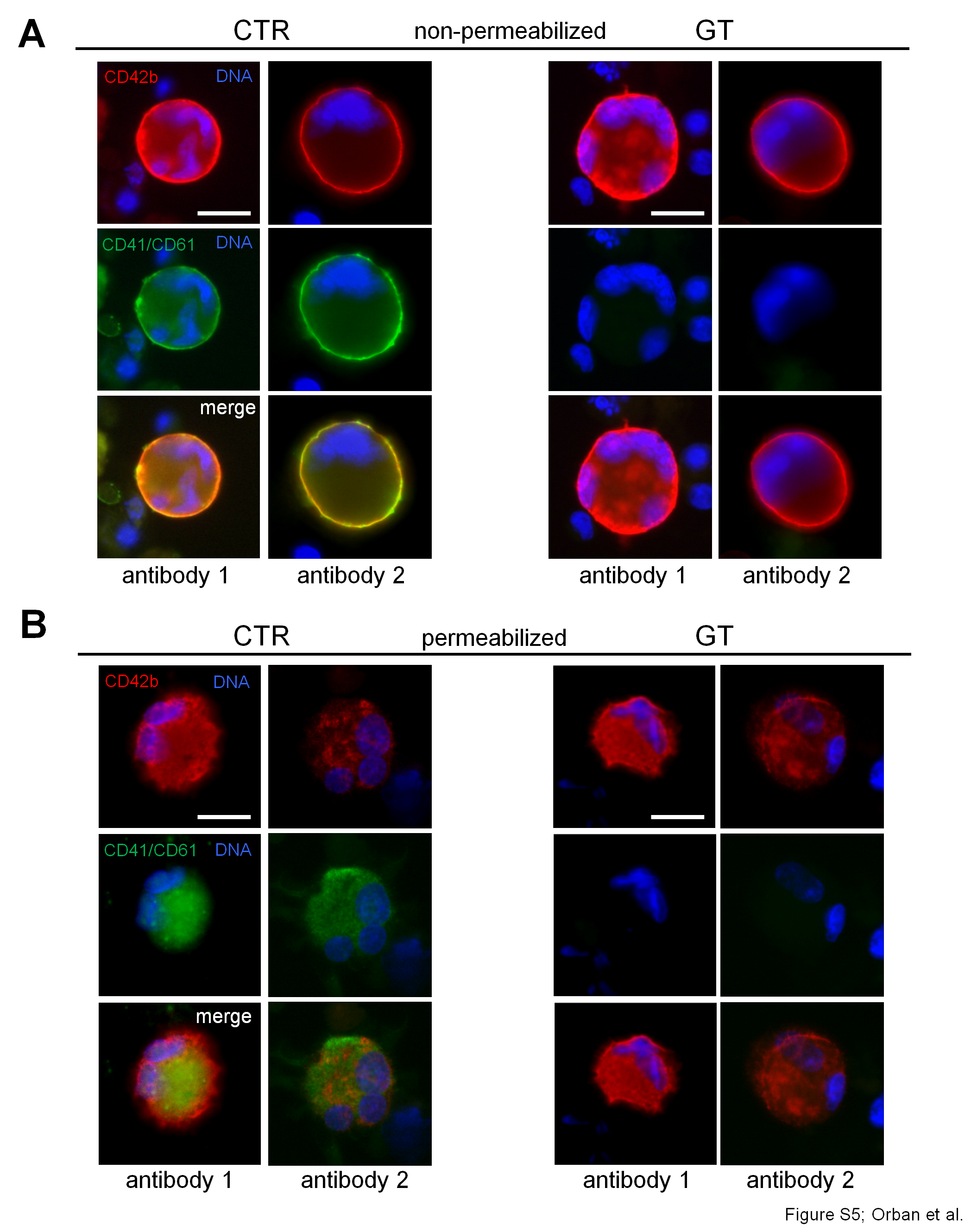

Supplement: S5 Fig — Cells were stained with anti-CD42b (red) and two different anti-CD41/CD61 (green; antibody 1 = HIP8, antibody 2 = P2) antibodies and DAPI (blue) with (A) or without (B) Triton-X100 permeabilization. All scale bars represent 20 μm. Representative images for each sample. (TIF) [file pone.0115978.s005.tif]

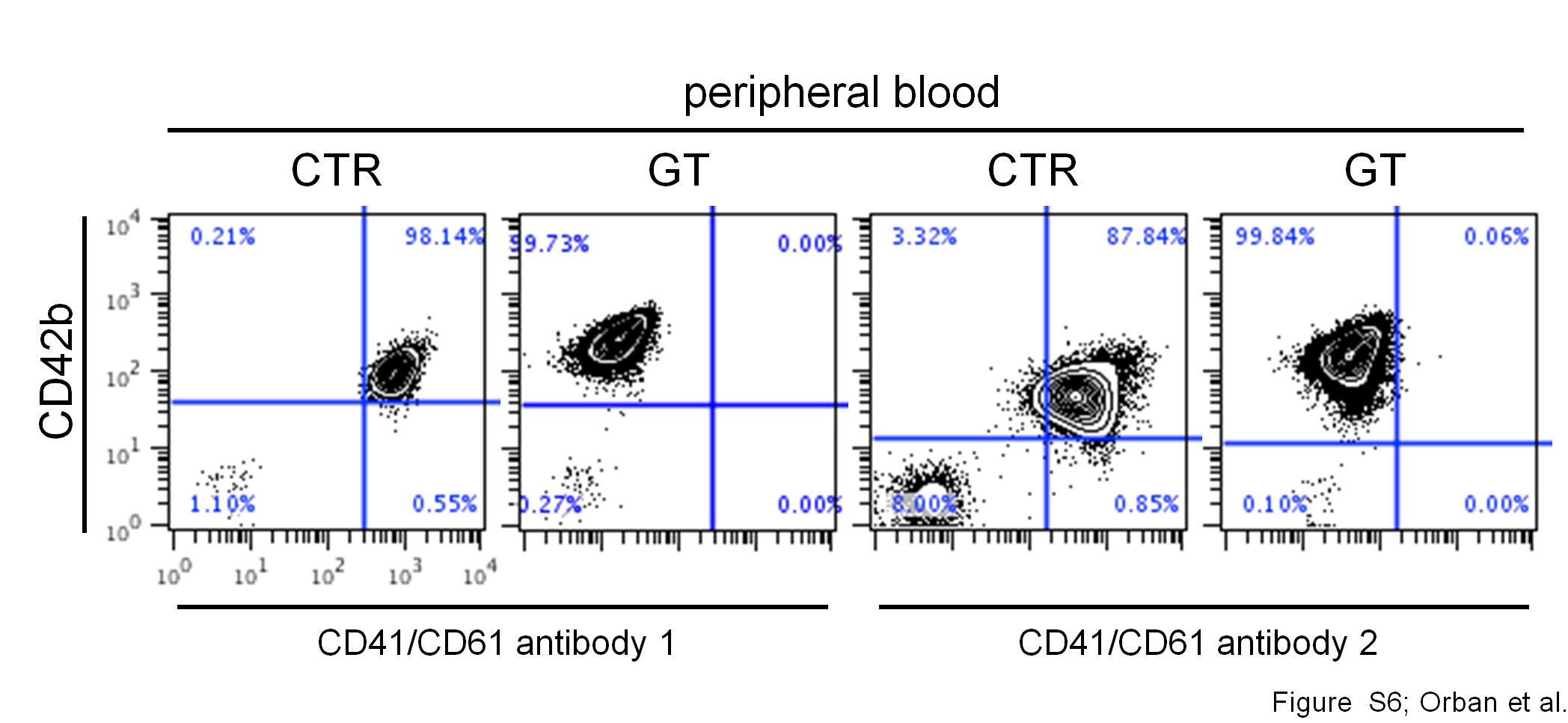

Supplement: S6 Fig — Peripheral blood platelets of CTR and GT were stained with anti-CD42b (y-axis) and two different anti-CD41/CD61 antibodies (x-axis; antibody 1 = clone HIP8; antibody 2 = clone P2) and DAPI (blue). (TIF) [file pone.0115978.s006.tif]

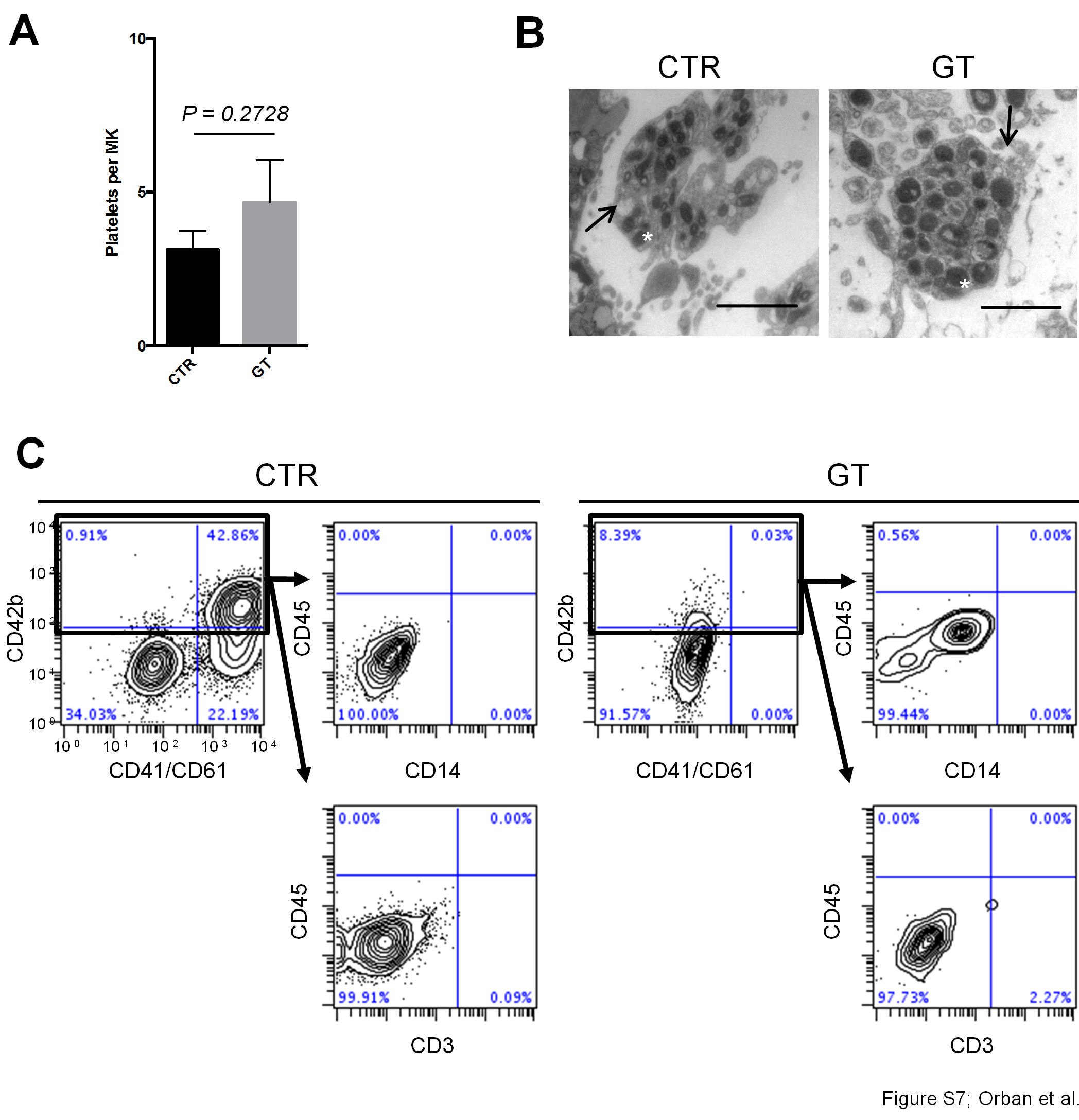

Supplement: S7 Fig — (A) Quantification of hiPSC-derived platelet yield per MK from GT and CTR (GT n = 11, CTR n = 15) by flow cytometry. Total number of platelets was divided by the number of MKs. P-value from unpaired t-test (B) Transmission electron microscopy images of hiPSC-derived platelets from GT and CTR show granules (asterisks) and the open canalicular system (arrows). Scale bar represents 2 μm. (C) Flow cytometry of CD3 and CD14 expression of hiPSC-derived platelets. HiPSC-derived cells of GT and CTR were stained with anti-CD42b, anti-CD41/CD61, anti-CD45, anti-CD3 and anti-CD14 antibodies. The FSC/SSC log gate of peripheral platelets was applied. For gating hierarchy see S10 Fig. Representative images for each sample. (TIF) [file pone.0115978.s007.tif]

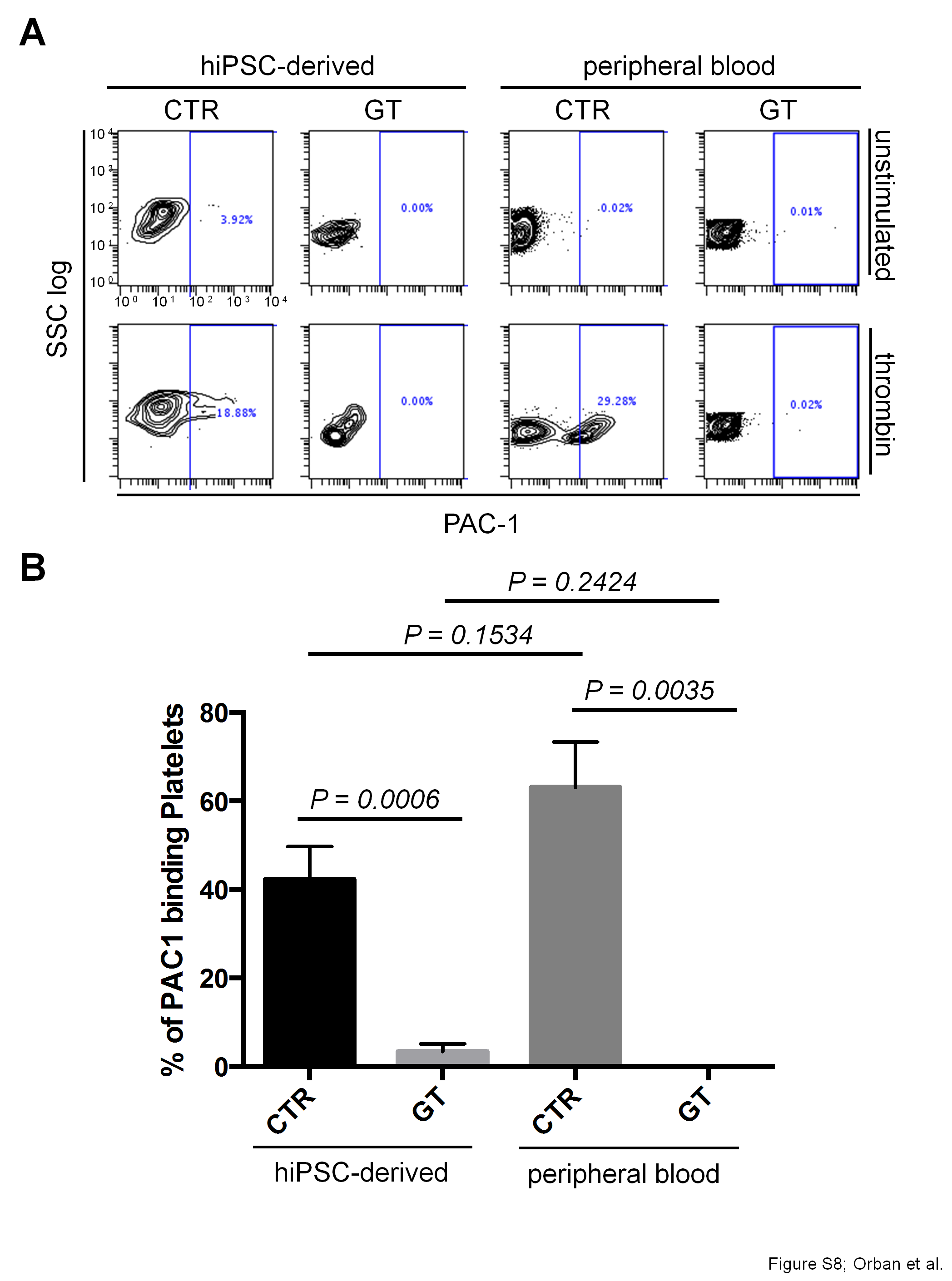

Supplement: S8 Fig — (A) HiPSC-derived and peripheral blood platelets were stained with PAC-1 and anti-CD42b antibodies in the absence (top) and presence (bottom) of thrombin. The FSC/SSC log gate of peripheral platelets was applied and further gated for CD42b positive cells. Representative images for each sample. For gating hierarchy see S10 Fig. (B) Quantification of PAC-1 binding after activation with ADP/TXA2. HiPSC-derived and peripheral platelets of CTR and GT were stained with anti-CD42b and PAC-1 antibodies and exposed to ADP/TXA2. The percentage of PAC-1+ of all CD42b+ platelets after ADP/TXA2 exposure is shown. P-values from unpaired t-tests. (TIF) [file pone.0115978.s008.tif]

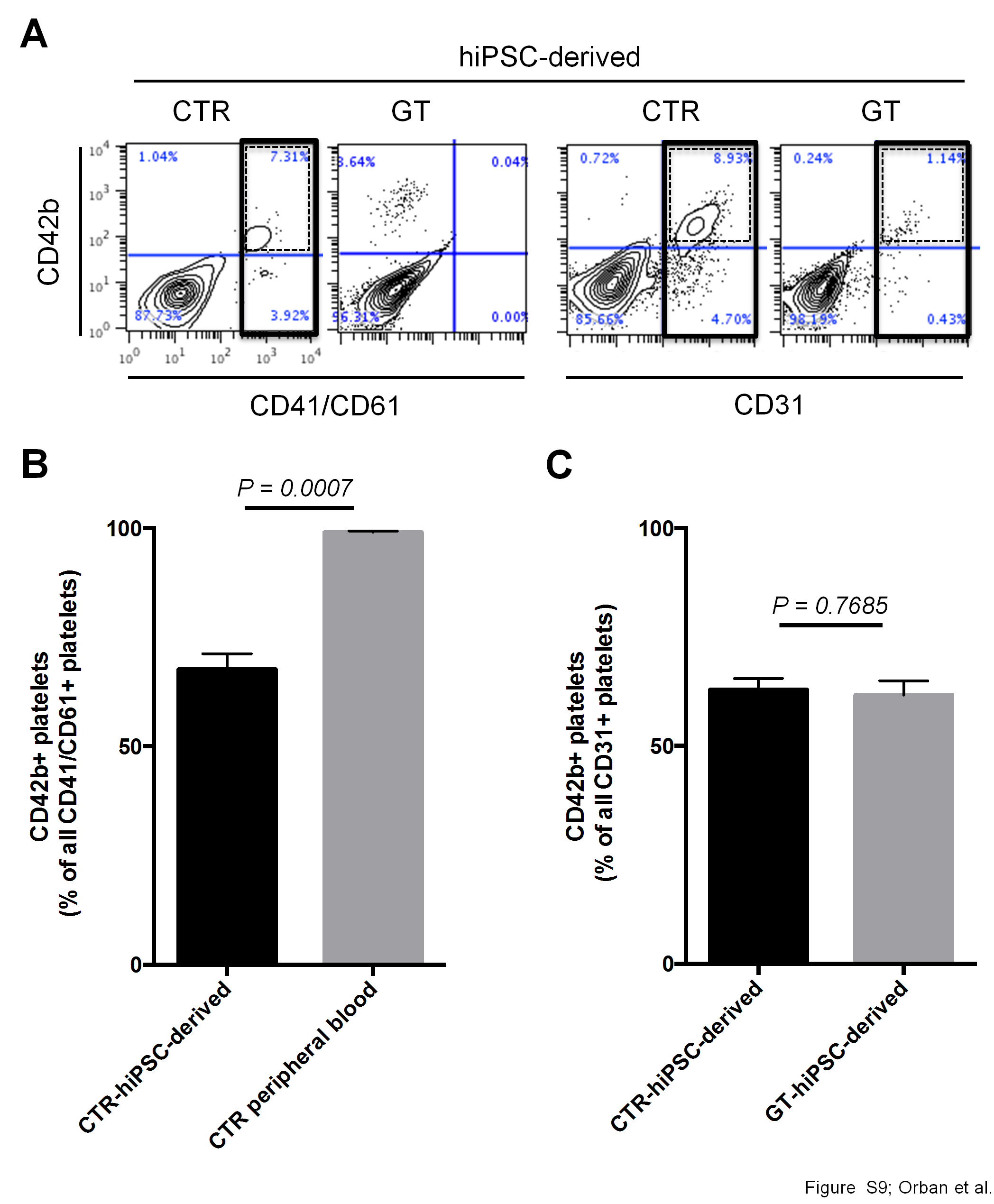

Supplement: S9 Fig — (A) HiPSC-derived platelets were stained with anti-CD42b (y-axis) and anti-CD41/CD61 or anti-CD31 (x-axis) antibodies. Continuous frames comprise either CD41/CD61+ or CD31+ platelets. Dashed frames comprise either CD42b and CD41/CD61 double positive or CD42b and CD31 double positive platelets. Representative images for each sample. For gating hierarchy see S10 Fig. (B) Quantification of the percentage of CD42b+ platelets of all CD41/CD61+ hiPSC-derived or peripheral blood platelets of CTR. (C) Quantification of the percentage of CD42b+ platelets of all CD31+ hiPSC-derived platelets of CTR and GT. P-values from unpaired t-tests. (TIF) [file pone.0115978.s009.tif]

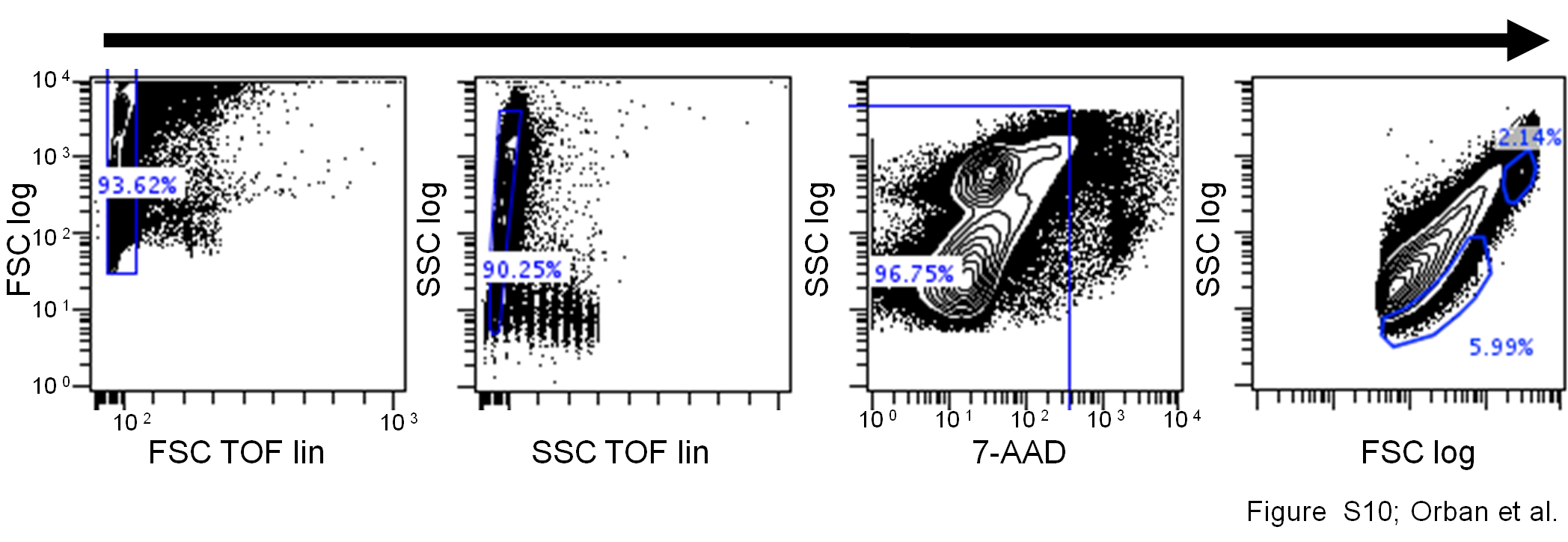

Supplement: S10 Fig — Flow cytometry gating hierarchy of hiPSC-derived MKs and platelets. Doublets of events were excluded with the FSC TOF (far left) and SSC TOF (left) parameters of the Gallios cytometer (Beckman Coulter). 7-AAD negative events (right) were further gated according to forward and sideward scatter parameters (far right). The FSC/SSC log gate of peripheral blood platelets was applied for analyzing platelets. A FSC high/SSC high population was selected for non-platelets or large cells including hiPSC-derived MKs and HPCs. (TIF) [file pone.0115978.s010.tif]
