# Supplementary material for: Functional Comparison of Induced Pluripotent Stem Cell- and Blood-Derived GPIIbIIIa Deficient Platelets
Source: PLoS One. 2015 Jan 21;10(1):e0115978. doi: 10.1371/journal.pone.0115978 (PMC4301811; doi:10.1371/journal.pone.0115978)
Supplement: S1 Methods — Supporting Material and Methods and Supporting References. (DOCX) [file pone.0115978.s011.docx]

**Supporting Material and Methods**

**Generation of hiPSCs from T-lymphocytes**

After approval from the ethical review board and written informed consent, PBMCs of a patient with type I GT and a gender- and age-matched control were isolated and T-lymphocytes were selectively activated for five days on CD3-coated plates with IL-2 (125 ng/ml) in AIM-V Medium (Life technologies). The cells were transfected with Sendai Virus coding for Oct3/4, Sox2, Klf4 and cMyc (CytoTune, Life technologies), after two days transferred onto mouse embryonic fibroblast (MEF) feeders and cultured in hiPSC medium (DMEM-F12, 20% serum knockout replacement, 0.1 mmol/l nonessential amino acids, 10 ng/ml basic fibroblast growth factor, 2 mmol/l L-Glutamine, 0.1 mmol/l beta-mercaptoethanol). Characterization of the hiPSCs was performed as previously described [1].

**Molecular genetic analysis**

Genomic DNA was isolated from ethylenediaminetetraacetic acid (EDTA) blood using Blood & Cell Culture DNA Kit (Qiagen). All exons from *ITGA2B* and *ITGB3* genes were amplified by PCR using intronic primers as previously described [2]. Exons were amplified and the resulting PCR product was separated in a 1% agarose gel, visualized by staining with GelRed (Biotium) and purified with the GFX PCR DNA and Gel Band Purification Kits (Amersham Biosciences). The purified DNA fragments were directly sequenced using PCR primers (Table S1).

**Generation of platelets and megakaryocytes from hiPSCs**

According to a protocol previously established [3], hiPSC colonies were removed from MEF feeders using a dissociation buffer (0.25 % trypsin, 1 mg/ml collagenase IV, 20 % KSR, 1 mmol/l CaCl_2_ in PBS), transferred onto irradiated C3H10T1/2 cells and differentiated with IMDM medium containing 10 mg/l insulin, 5.5 mg/l transferring, 6.7 mg/ml selenium, 2 mmol/l L–glutamine, 15% fetal bovine serum (all Gibco), 0.45 μmol/l α-monothioglycerol (Sigma-Aldrich), 50 μg/ml ascorbic acid (Sigma-Aldrich) and 20 ng/ml recombinant human vascular endothelial growth factor (Invitrogen). On day 15, hiPSC-sacs were disrupted with a cell scraper, crushed with a pipette and passed through a 40 µm cell strainer (BD Falcon). The yielded cells were transferred onto irradiated C3H10T1/2 cells and cultured in the same medium without vascular endothelia growth factor containing 100 ng/ml human TPO (R&D), 50 ng/ml human SCF (R&D), and 25 U/ml heparin (Sigma-Aldrich). Medium was changed every 3 days. From days 24 to 30 floating cells were collected for analysis.

**Immunostaining**

Cytospin slides with MKs and platelets were prepared for immunofluorescence studies. Cells were fixed (2% paraformaldehyde/PBS, room temperature, 10 minutes), optionally permeabilized in 0.1% Triton X-100 and stained with FITC conjugated anti-human CD41/CD61 (BD; 1:100 dilution) and PE conjugated anti-human CD42b (Beckman Coulter) monoclonal antibodies (1:50 dilution). Images were taken through a 63x oil immersion objective with a DFC 320 camera on a DMI 6000B microscope (Leica).

**Quantitative real-time PCR (qRT-PCR) and reverse transcription PCR (RT-PCR)**

Total mRNA was isolated from T-lymphocytes, iPSC clones and EBs using the Stratagene Absolutely RNA kit and 1 μg was used to synthesize cDNA using the High-Capacity cDNA Reverse Transcription kit (Applied Biosystems). Gene expression was quantified by qRTPCR using 1 μl of the RT reaction and the Power SYBR Green PCR Master Mix (Applied Biosystems). Gene expression levels were normalized to GAPDH. A list of the primers is provided Table S1.

**Flow cytometry, platelet activation, spreading and fibrinogen binding quantification**

Cells from supernatant of day 24 to 30 were spun down for 10 min at 1000 g. Fresh peripheral blood containing acid-citrate-dextrose was centrifuged for 5 min at 100 g, and the resultant supernatant centrifuged for 10 min at 400 g. Pellets from all specimens were resuspended in 200 µl 1% BSA/PBS and stained with 7-AAD or fluorochrome-conjugated monoclonal antibodies (BD and Beckman Coulter) for 30 min at room temperature (Table S2). For platelet activation, the pellet was resuspended in 200 µl of Tyrode’s buffer (pH 7.4) with 10 mmol/l HEPES (Life Technologies), 1 mg/ml Glucose (Sigma-Aldrich), 1 mg/ml fatty acid free BSA (Sigma-Aldrich), 1 mmol/l CaCl_2_ and 1 mmol/l MgCl_2_. For PAC1-binding, cells were treated with 5 µmol/l adenosine diphosphate (ADP, Sigma-Aldrich) and 2.8 µmol/l thromboxane A_2_ (TXA_2_, Enzo) or 1 U/ml thrombin for 20 min at 37 °C and then stained with anti-CD42b and PAC-1 (BD; 1:50 dilution) for 30 min at room temperature. Flow cytometry data were acquired using a Gallios flow cytometer (Beckman Coulter) and analyzed with Cytobank [4]. Differential interference contrast (DIC) microscopy images of platelets spreading on fibrinogen-coated wells after treatment with 5 µmol/l ADP and 2.4 µmol/l TXA were recorded at indicated time intervals using an inverted IX 70 microscope with an UPlan 40x/1.00 or 20x oil-immersion objective (Olympus), equipped with a custom-built climate chamber (5% CO2, 37°C, humidified) [5]. Images were captured with a FluoView II camera controlled by Cell D software (Olympus). For quantification of fibrinogen binding, wells were coated with 100 µg/ml fibrinogen for 1 h at 37 °C and washed once with PBS before use. Cells of supernatant were resuspended in 500 µl Tyrode’s buffer (pH 7.4), stained with rhodamine (1:40 dilution) for 15 min and plated on fibrinogen-coated wells. 50 µmol/l ADP, 2.8 µmol/l TXA_2_, 1 mmol/l CaCl_2_ and 1 mmol/l MgCl_2_ were added and incubated for 20 min at 37°C. After washing the wells twice with PBS, attached cells were imaged with a DFC 320 camera on a DMI 6000B microscope (Leica)**.**

**Transmission electron microscopy**

Cells from supernatant of day 24 to 30 were spun down for 10 min at 1000 g. The fractions were fixed isoosmotically with 2.5% glutardialdehyde in fixative buffer (75 mM sodium cacodylate, 75 mM NaCl, 2 mM MgCl2, pH 7.0), for 1 h at room temperature. Afterwards, samples were rinsed several times in fixative buffer and post-fixed at room temperature for 1 h with 1% osmium tetroxide in fixative buffer. After two washing steps in distilled water, the cells were stained en bloc for 30 min with 1% uranyl acetate in 20% acetone. Dehydration was performed with a graded acetone series. Samples were then infiltrated and embedded in Spurr’s low-viscosity resin. Ultrathin sections were cut with a diamond knife and mounted onto collodion coated copper grids. The sections were post-stained with aqueous lead citrate (100 mM, pH 13.0). Transmission electron micrographs were taken with an EM 912 electron microscope (Zeiss, Oberkochen, Germany) equipped with an integrated OMEGA energy filter operated at 80 kV in the zero loss mode.

**Supporting References**

1. Jung CB, Moretti A, Mederos y Schnitzler M, Iop L, Storch U, et al. (2012) Dantrolene rescues arrhythmogenic RYR2 defect in a patient-specific stem cell model of catecholaminergic polymorphic ventricular tachycardia. EMBO Mol Med 4: 180-91.
2. Vannier C, Behnisch W, Bartsch I, Sandrock K, Ertle F, et al. (2010) Novel homozygous mutation (c.175delG) in platelet glycoprotein ITGA2B gene as cause of Glanzmann's thrombasthenia type I. Klin Padiatr 222: 150-153.
3. Takayama N, Eto K (2012) In vitro generation of megakaryocytes and platelets from human embryonic stem cells and induced pluripotent stem cells. Methods Mol Biol  788: 205-17.
4. Kotecha N, Krutzik PO, Irish JM (2010) Web-based Analysis and Publication of Flow Cytometry Experiments. Curr Protoc Cytom; Chapter 10:Unit10.
5. Zhang L, Orban M, Lorenz M, Barocke V, Braun D, et al. (2012) A novel role of sphingosine 1-phosphate receptor S1pr1 in mouse thrombopoiesis. J Exp Med 209: 2165-81.
