# Supplementary material for: Functional Comparison of Induced Pluripotent Stem Cell- and Blood-Derived GPIIbIIIa Deficient Platelets
Source: PLoS One. 2015 Jan 21;10(1):e0115978. doi: 10.1371/journal.pone.0115978 (PMC4301811; doi:10.1371/journal.pone.0115978)
Supplement: S1 Table — PCR-Primers used for sequencing of exons containing the mutations in the ITGA2B gene. (DOCX) [file pone.0115978.s019.docx]

**Supporting Table**

**Table S1. PCR-Primers used for sequencing of exons containing the mutations in the *ITGA2B* gene.**

**Accession no. NG_008331**

| **Name** | **Sequence** |
| --- | --- |
| *ITGA2B* E19fw | 5´-CCCAAACCTCCAAATAAACCC-3’ |
| *ITGA2B* E20rev | 5´-CCATTAGCAAGTATTCCTCCTCCA-3´ |
| *ITGA2B* E23fw | 5´-AGCTCTCTCTGAACTCTCTAA-3´ |
| *ITGA2B* E26rev | 5´-ACCCCTCTCCCTCCTCCCATCC-3´ |
