# Supplementary material for: Functional Comparison of Induced Pluripotent Stem Cell- and Blood-Derived GPIIbIIIa Deficient Platelets
Source: PLoS One. 2015 Jan 21;10(1):e0115978. doi: 10.1371/journal.pone.0115978 (PMC4301811; doi:10.1371/journal.pone.0115978)
Supplement: S2 Table — Antibodies/Flow Cytometry Dye. (DOCX) [file pone.0115978.s020.docx]

**Supporting Table**

**Table S2.** **Antibodies/Flow Cytometry Dye**

| Antigen | Clone | Fluorochrome | Manufacturer | Cat. # | Dilution |
| --- | --- | --- | --- | --- | --- |
| CD3 | UCHT1 | FITC | Beckman Coulter | A07746 | 1:50 |
| CD14 | RMO52 | PE | Beckman Coulter | IM0650U | 1:50 |
| CD31 | 9G11 | PE | R&D | FAB3567P | 1:50 |
| CD34 | 581 | FITC | BD Pharmingen | 560942 | 1:100 |
|  | 581 | APC | BD Pharmingen | 555824 | 1:100 |
| CD41 (in complex with CD61) | HIP8 | FITC | BD Pharmingen | 555466 | 1:100 |
|  | P2 | PC7 | Beckman Coulter | IM0649U | 1:50 |
| CD41 (α-chain of CD41) | SZ22 | FITC | Beckman Coulter | IM1756U | 1:50 |
| CD42b | SZ2 | PE | Beckman Coulter | IM1417U | 1:50  1:50 |
|  | SZ2 | FITC | Beckman Coulter | IM0648U |  |
| CD45 | J33 | Pacific Blue | Beckman Coulter | A74763 | 1:50 |
|  | HI30 | PerCP-Cy5.5 | BD Pharmingen | 564105 | 1:100 |
| CD61 | SZ21 | FITC | Beckman Coulter | IM1758 | 1:50 |
| FOXA2 (HNF-3β) | polyclonal | - | Santa Cruz Biotechnology | sc-6554 | 1:100 |
| NANOG | polyclonal | - | Abcam | ab80892 | 1:500 |
| PAC-1 | PAC-1 | FITC | BD Pharmingen | 340507 | 1:100 |
| p75 NGF Receptor | MLR2 | FITC | Pierce | MA1-18421 | 1:100 |
| Sendai Virus (SeV) | polyclonal | - | MBL | PD029 | 1:100 |
| CARDIAC TROPONIN T | 13-11 | - | Pierce | MA5-12960 | 1:100 |
| TRA-1 81 | TRA1 81 | AlexaFluor-488 | BD Pharmingen | 560174 | 1:20 |
| 7-AAD | - | - | Beckman Coulter | A07704 | 1:50 |
